# Projected bioclimatic distributions in Nearctic *Bovidae* signal the potential for reduced overlap with protected areas

**DOI:** 10.1002/ece3.9189

**Published:** 2022-08-11

**Authors:** Christian John, Eric Post

**Affiliations:** ^1^ Department of Wildlife, Fish, and Conservation Biology University of California Davis California USA

**Keywords:** Bovidae, climate change, conservation, MaxEnt, range shift, species distribution modeling

## Abstract

Assumptions about factors such as climate in shaping species' realized and potential distributions underlie much of conservation planning and wildlife management. Climate and climatic change lead to shifts in species distributions through both direct and indirect ecological pressures. Distributional shifts may be particularly important if range overlap is altered between interacting species, or between species and protected areas. The cattle family (*Bovidae*) represents a culturally, economically, and ecologically important taxon that occupies many of the world's rangelands. In contemporary North America, five wild bovid species inhabit deserts, prairies, mountains, and tundra from Mexico to Greenland. Here, we aim to understand how future climate change will modify environmental characteristics associated with North American bovid species relative to the distribution of extant protected areas. We fit species distribution models for each species to climate, topography, and land cover data using observations from a citizen science dataset. We then projected modeled distributions to the end of the 21st century for each bovid species under two scenarios of anticipated climate change. Modeling results suggest that suitable habitat will shift inconsistently across species and that such shifts will lead to species‐specific variation in overlap between potential habitat and existing protected areas. Furthermore, projected overlap with protected areas was sensitive to the warming scenario under consideration, with diminished realized protected area under greater warming. Conservation priorities and designation of new protected areas should account for ecological consequences of climate change.

## Graphical abstract

Members of the cattle family (Bovidae) in North America face considerable change under projected climate change. In this article, we describe ecological niche models fit to occurrence data of five wild bovid species and predict current and future distribution of bioclimatic factors characterizing species occurrence under two climate change scenarios. We measure the degree of overlap between modeled distributions and currently protected areas, finding that ecological niche space is expected to shift most dramatically among more northerly species, leading to larger reductions in realized protected space.

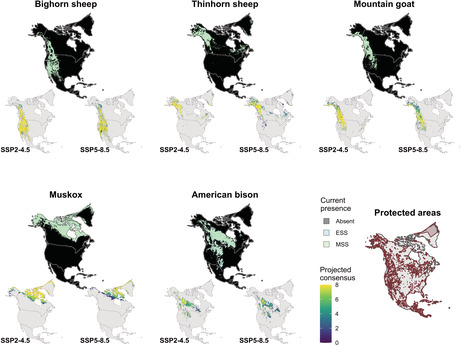

## 1 INTRODUCTION

Elevational and latitudinal shifts in species' ranges constitute widely documented ecological responses to climate change (Büntgen et al., 2017; Chen et al., 2011; Williams & Blois, 2018). Through both direct (e.g., thermal stress) and indirect (e.g., temperature‐mediated natural enemy activity) mechanisms, climate shapes species distributions across local, regional, and global scales (Araújo & Luoto, 2007). As ongoing human pressure further shapes contemporary species distributions (Faurby & Araújo, 2018; Laliberte & Ripple, 2004), identifying factors associated with species presence and measuring how these factors will change lends insight on how potential species distributions may shift in the coming decades. Effective conservation planning, therefore, relies on well‐defined forecasts of the change in species distribution (Rodríguez et al., 2007). Yet, for many species, the extent to which future distributions will overlap with existing protected areas remains unresolved (IPBES, 2019).

The designation of effective protected areas requires balancing the immediate needs of imperiled species with anticipated conditions decades or centuries into the future. Although the establishment of protected areas has increased dramatically over the past century (Watson et al., 2014), the density, area, and governance of protected areas vary considerably across space (Bingham et al., 2019; UNEP‐WCMW and IUCN, 2021). As conserved spaces continue to be planned and adopted, formal analyses of interactions among climate and geographical factors governing species distributions and projected changes in them will aid in the prioritization of areas to protect (Monzón et al., 2011; Scridel et al., 2021; Sierra‐Morales et al., 2021). Biotic interactions may yet further control species distributions, especially for herbivores that specialize on particular food resources (e.g., Beumer et al., 2019). Thus, effective conservation planning will take into account not only future change in temperature and precipitation, but also shifts in vegetation distributions and land cover types.

In North America, the mammalian family *Bovidae* is represented by five extant species: bighorn sheep (*Ovis canadensis*), thinhorn sheep (*Ovis dalli*), North American bison (*Bison bison*), mountain goat (*Oreamnos americanus*), and muskox (*Ovibos moschatus*). These species constitute a broad‐ranging phylogeographic clade that survived marked warming at the end of the Pleistocene. Today, they occupy deserts, prairies, tundra, and alpine zones across the Nearctic (Castelló, 2016). The bovid species of North America accumulated a legacy of hunting, introduced disease, and human development, leading to shifts in abundance, migratory propensity, and distributions.

Here, we fit and project Ecological Niche Models (ENMs) for Nearctic bovid species under two scenarios of anticipated climate change generated using occurrence data from a public database of species observations. We relate modeled current and future species distributions to existing protected areas, with the goals of identifying how environmental parameters may shift in the coming decades, and how well current protected areas align with modeled distributions. We discuss our modeling results in the context of other work on conservation and spatial variation in wild bovids.

## 2 METHODS

### 2.1 Species presence data

Species presence data were downloaded from the Global Biodiversity Information Facility (GBIF, 2022). This database includes species presence observations from museum collections, university records, and citizen science contributions. Presence data were extracted using the ‘rgbif’ library in R v.3.6.1 (Chamberlain et al., 2021), with GBIF taxon key associated with each of the five North American bovids (*O. canadensis*, 2441119; *O. dalli*, 2441118; *O. americanus*, 2441151; *O. moschatus*, 2441108; and *B. bison*, 2441176), as well as the remaining North American members of Artiodactyla (*Antilocapra americana*, 2440902; *Odocoileus hemionus*, 2440974; *Odocoileus virginianus*, 2440965; *Cervus canadensis*, 8600904; *Alces alces*, 4262283; *Rangifer tarandus*, 5220114; and *Dicotyles tajacu*, 2440996). Occurrence data were sent through a cleaning process to remove biased, uninformative, or inappropriate observations (for a full description of removed observations, see “Biodiversity data” in Table S1). First, points with missing geographic information were censored. Next, observations outside of North America were removed, as were cases where observation locations did not correspond with observation country. Records with no associated observation date, and records with observation date prior to 1980, were removed. Finally, irrelevant observation locations (e.g., bighorn sheep at the Chicago Zoo) were removed.

Data generated through citizen science collection face concerns over validity and sampling bias (Beck et al., 2013; Yesson et al., 2007). The dataset we used constitutes a set of charismatic, easily identified species, in a generally well‐sampled geographic region (see Table S1, Biodiversity Data). Because presence‐only species distribution models are sensitive to spatial biases in sampling effort (Phillips et al., 2009), we used occurrence data from the full set of North American even‐toed ungulates to generate a sampling bias grid, which was used during the background data generation (described below). Furthermore, we coarsened the resolution of the predictor dataset to accommodate uncertainty in observation location. However, our efforts to control for biases in species presence data limit the resolving power of species distribution, and we were thus unable to account for the effects of microclimate (e.g., Lembrechts et al., 2019) in our models, or incorporate anticipated fine‐scale change in our projections.

### 2.2 Climate, land cover, and topography data

Historical and projected Worldclim v. 2.1 data (Fick & Hijmans, 2017), present and future GCAM land cover data (Chen et al., 2020), and North America Elevation GRID data (available at https://www.sciencebase.gov/catalog/item/4fb5495ee4b04cb937751d6d) were used as baseline environmental covariates. All predictors were coarsened to 6 × 6 km pixels in an equal‐area projection to accommodate spatial uncertainty and match the resolution of the coarsest predictor data product in species occurrence data using bilinear interpolation.

Current and future climate data were accessed from the Worldclim v. 2.1 dataset. We selected data generated from all eight available global climate models (GCMs; BCC‐CSM2‐MR, CanESM5, CNRM‐CM6‐1, CNRM‐ESM2‐1, IPSL‐CM6A‐LR, MIROC‐ES2L, MIROC6, and MRI‐ESM2‐0) under two shared socioeconomic pathways (SSPs; SSP2–4.5 and SSP5–8.5) for the period 2081–2100. SSPs were adopted with the CMIP6 models and incorporated socioeconomic growth with the previously used representative concentration pathways (Riahi et al., 2017). SSP2 reflects a future with moderate development, on track with historical growth and inequality, but with reduced dependence on fossil fuels, whereas SSP5 reflects a future with accelerating socioeconomic development and reduced global inequality, but with a heavy reliance on fossil fuels. SSP2–4.5 predicts about 3°C warming by the end of this century, while SSP5–8.5 predicts about 5°C warming relative to the 1850–1900 average (Tebaldi et al., 2021). Data on future conditions were re‐centered and transformed according to the approach described for historical data above.

Current and future (2081–2100) land use/land cover data were accessed from the GCAM Demeter land use dataset (Chen et al., 2020). GCAM data are reported by cover type on a fractional scale from 0 to 100, where 100 indicates the pixel is saturated by that type. We aggregated each of the GCAM tree cover types into their respective biome (PFT4 and 6; 1, 5, and 7; and 2, 3, and 8 representing tropical, temperate, and boreal trees, respectively), and PFT15–30 into an umbrella category, “Agriculture,” to reduce the size of the candidate predictor pool. Thus, from the GCAM data we included 14 vegetation layers, an agriculture layer, a barren layer, and an urban layer. We used the SSP1–2.6 2015 model to index current land cover conditions. Because GCAM data are not available for the same CMIP6 models as Worldclim, we condensed the five available models of land use futures into their respective SSP scenarios (SSP2–4.5 and SSP5–8.5) by taking the mean value of each fractional land cover type for each pixel across the five available models. The “current” SSP1–2.6 scenario was also condensed from the five available models.

To account for topographic constraints on species distribution, we included elevation and terrain ruggedness as predictors. Terrain ruggedness (TRI) was calculated following standard gdal protocols (GDAL/OGR contributors, 2021). Finally, elevation and TRI were centered by subtracting the mean layer value from all grid cells within each layer. Topography data were treated as static, and therefore, the same topography products were used for present and future (2081–2100) datasets.

### 2.3 Vector spatial data

Land boundaries of North America were extracted from the rnaturalearth::ne_countries() dataset (South, 2017). The periphery of the Greenland Inland Ice Sheet was delineated by vectorizing all cells classified as “ice” in the raster version of the Circumpolar Arctic Vegetation Map (Raynolds et al., 2019). Protected area boundaries were identified using the World Database of Protected Areas (UNEP‐WCMW and IUCN, 2021) and filtered to include only polygons with area >100 km^2^.

### 2.4 Statistical modeling

Complete details on overview, data, model design, assessment, and prediction (ODMAP; Zurell et al., 2020) are available in Table S1. MaxEnt v. 3.4.3 models (Phillips et al., 2021) were fit to the presence and background locations for each bovid species. We used the ‘SDMtune’ library (Vignali et al., 2020) to fit, evaluate, and generate predictions with MaxEnt models. For each species, a MaxEnt model was constructed using the following approach: Occurrence records were spatially thinned to a radius of 6 km. A bias grid was generated using occurrence data from all North American artiodactyl species to account for sampling bias in occurrence data (Phillips et al., 2009). We assumed that sampling bias was equivalent across Artiodactyla, given that they are large, charismatic, and easily identifiable, and therefore used one bias grid for the continent. The bias grid was calculated by generating a continental raster with 6 × 6 km pixel resolution, identifying all pixels containing artiodactyl species occurrences, and applying a 2‐dimensional kernel density estimator with a normal reference bandwidth. Ten thousand background points were randomly sampled from the bias grid in lieu of absence data for model fitting for each species. Occurrence and background data were subdivided into 60% training, 20% validation, and 20% testing partitions. Naïve MaxEnt models were fit with training data and spatial cross‐validation using the checkerboard1 function in the R package ENMeval (Kass et al., 2021). To minimize model complexity and reduce the likelihood of overfitting, we considered only linear and quadratic feature classes (Elith et al., 2011). We assumed no a priori knowledge of factors associated with species presence and, therefore, included all 19 bioclimatic variables, all topographic covariates, and all land cover indices in the naïve models. A data‐driven variable selection procedure was then employed to remove highly correlated predictor variables, based on a Spearman correlation threshold of 0.7 (Vignali et al., 2020). After removing correlated predictors, models were optimized for complexity using a genetic algorithm to identify the most robust combination of model hyperparameters. We considered regularization multipliers between 0.5 (most complex) and 10 (least complex) and linear as well as linear+quadratic feature classes. Finally, we removed non‐important variables from the optimized models to maximize parsimony using a leave‐one‐out jackknife test. We refer to these optimized models with selected variables as the “final model” for each species. Final model reports were generated for each species (summarized in Figure S1).

Species distributions were predicted using final models and three raster stacks: “current” conditions defined by the training data, and two future scenarios (SSP2–4.5 and SSP5–8.5), both for the period spanning 2081–2100. Because MaxEnt models generate continuous prediction surfaces, model‐specific response thresholds were used to differentiate between predicted “presence” and “absence.” We used two thresholds (Liu et al., 2013): one with equal model sensitivity and specificity (ESS) and one which maximized the sum of sensitivity and specificity (MSS). For each future SSP scenario, model consensus was calculated as the sum of the MaxEnt model predictions under each GCM that were above the MSS threshold, based on a comparison between the two thresholds under current conditions revealing few differences except for a more constrained bison range using MSS. Correlative distribution modeling approaches such as MaxEnt are limited by uncertainty in future conditions, non‐analog conditions, and exclusion of endogenous factors that may allow species to adapt or tolerate future change (Dawson et al., 2011). Uncertainty in climate forecasts was accounted for by compositing modeled species distributions across environmental covariates predicted under a suite of GCM models. To account for non‐analog conditions, we applied a clamping procedure to prevent projecting results outside the range of conditions present during model training. We also generated multivariate environmental similarity surfaces (“MESS grids”) and limited predictions to areas with positive MESS values (Elith et al., 2010). MESS grids were calculated using the R package dismo instantiation of ‘mess()’ with all continuous predictors in the dataset and are shown in Table S1. We were unable to account for species' adaptive potential and thus limit our interpretation of the results below to anticipated change in distribution of environmental characteristics associated with bovid species presence, rather than distribution of bovid species themselves.

Comparisons among species of land area, range elevation, range latitude, and realized protected area were calculated by taking the mean value of current and projected data layers grouped by species and SSP. Standard errors of mean projected range measurements were calculated by treating GCM as a replicate. All analyses were performed in R v. 4.1.2 (R Core Team, 2019).

## 3 RESULTS

We accessed 32,999 North American bovid records from GBIF. We removed 14,514 observations during data quality checks and 14,927 during data thinning, leaving 3558 records for model fitting. Within the cleaned, thinned dataset, bighorn sheep were represented by 1915 records, thinhorn sheep by 218 records, mountain goats by 659 records, muskoxen by 218 records, and North American bison by 519 records.

**FIGURE 1:**
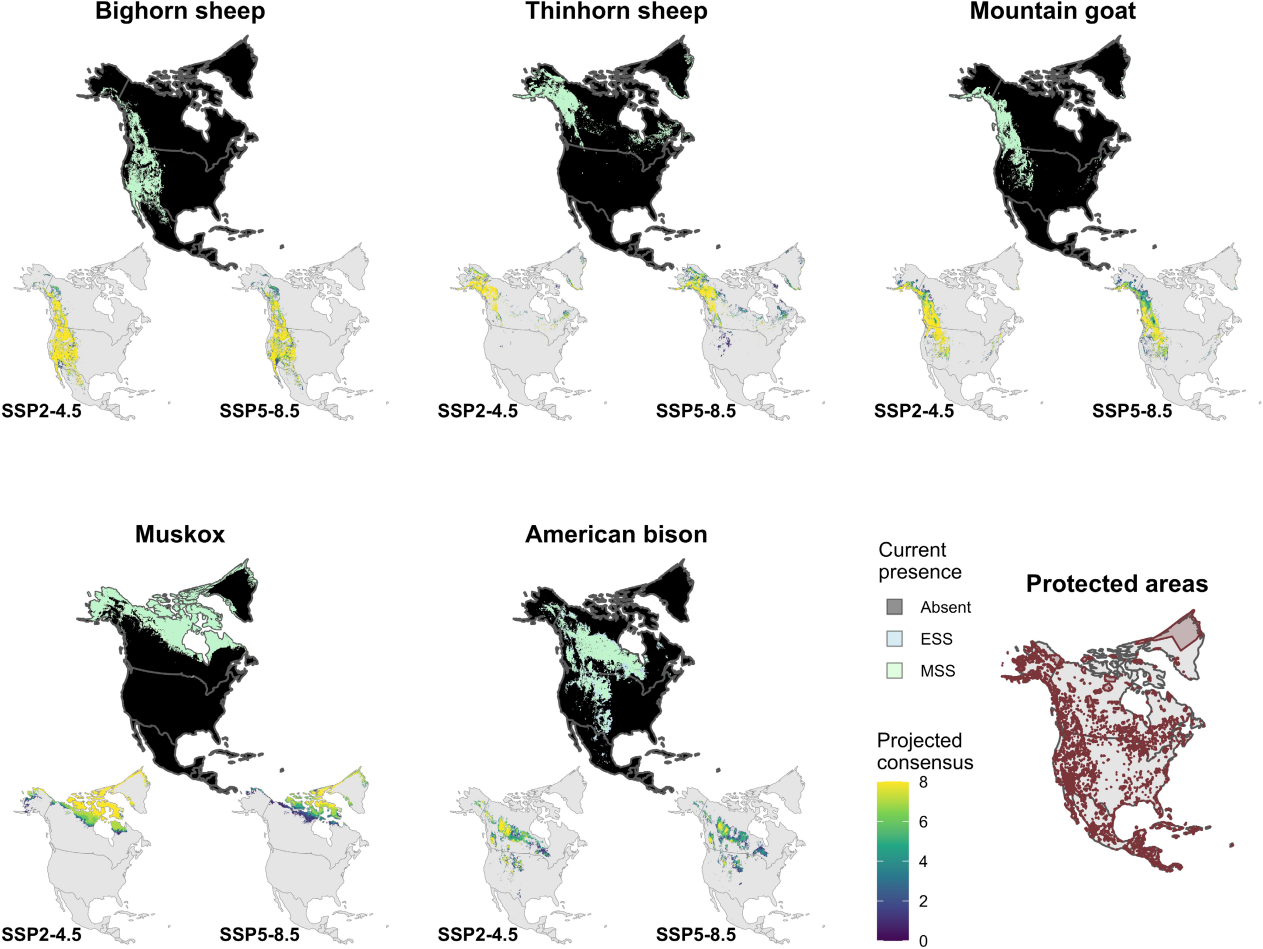
Predicted current potential habitat (top subplots) and consensus future potential habitat under future conditions in 2081–2100 modeled using two SSPs (bottom subplots) for each Nearctic bovid species. For the current plots, predicted potential habitat is indicated by pale blue (for the ESS threshold) and pale green (for the MSS threshold). For the consensus plots, the fill value increases in intensity with increasing predicted suitability across GCMs (using the MSS threshold). Protected areas indicated by merlot polygons, data from (UNEP‐WCMW and IUCN, 2021).

In general, modeled potential habitat shifted in response to projected climate change in 2081–2100 (Figure 1). Modeled future habitat covered less area under the SSP5–8.5 scenario than under the SSP2–4.5 scenario for all species except thinhorn sheep (Table 1). Projected change in the surface area of modeled habitat was inconsistent across species, but with a trend of increasing change at higher latitudes (Table 1). For example, over a quarter of modeled potential habitat space is expected to be lost for thinhorn sheep by 2100 regardless of the SSP, while the projected change for bighorn sheep is less coherent. The total area of modeled potential habitat was never consistently higher under both scenarios for any species (although modeled potential habitat increased slightly under SSP2–4.5 for bighorn sheep and mountain goats).

**TABLE 1:** Surface area of modeled species distributions under current (1970–2000) and projected future (2081–2100) conditions, expressed in millions of km^2^.

Species	Current	SSP2–4.5	SSP5–8.5
Bighorn sheep	2.46	2.53 ± 0.04	2.18 ± 0.09
Thinhorn sheep	2.20	1.21 ± 0.04	1.50 ± 0.09
Mountain goat	1.86	1.89 ± 0.04	1.65 ± 0.08
Muskox	5.00	2.77 ± 0.16	1.62 ± 0.21
American bison	3.34	1.22 ± 0.14	1.01 ± 0.18

Projected elevational range shifts were variable among species (Table S2‐S3). Whereas projections for bighorn sheep featured marginal elevational change (current mean elevation = 1527 m; SSP2–4.5 mean elevation = 1537 ± 5 m; SSP5–8.5 mean elevation = 1583 ± 7 m), stronger elevational contraction was evident for thinhorn sheep (current mean elevation = 826 m; SSP2–4.5 mean elevation = 932 ± 17 m; SSP5–8.5 mean elevation = 934 ± 44 m). Projected latitudinal range shifts were similarly variable among species. For example, modeled muskox habitat faces a significant northward contraction due to limited available land area further north (current mean latitude = 68.1°N; SSP2–4.5 mean latitude = 71.7 ± 0.4°N; SSP5–8.5 mean latitude = 74.5 ± 0.5°N), while modeled mountain goat habitat shifts slightly southward (current mean latitude = 53.1°N; SSP2–4.5 mean latitude = 52.2 ± 0.4°N; SSP5–8.5 mean latitude = 51.8 ± 0.9°N).

**FIGURE 2:**
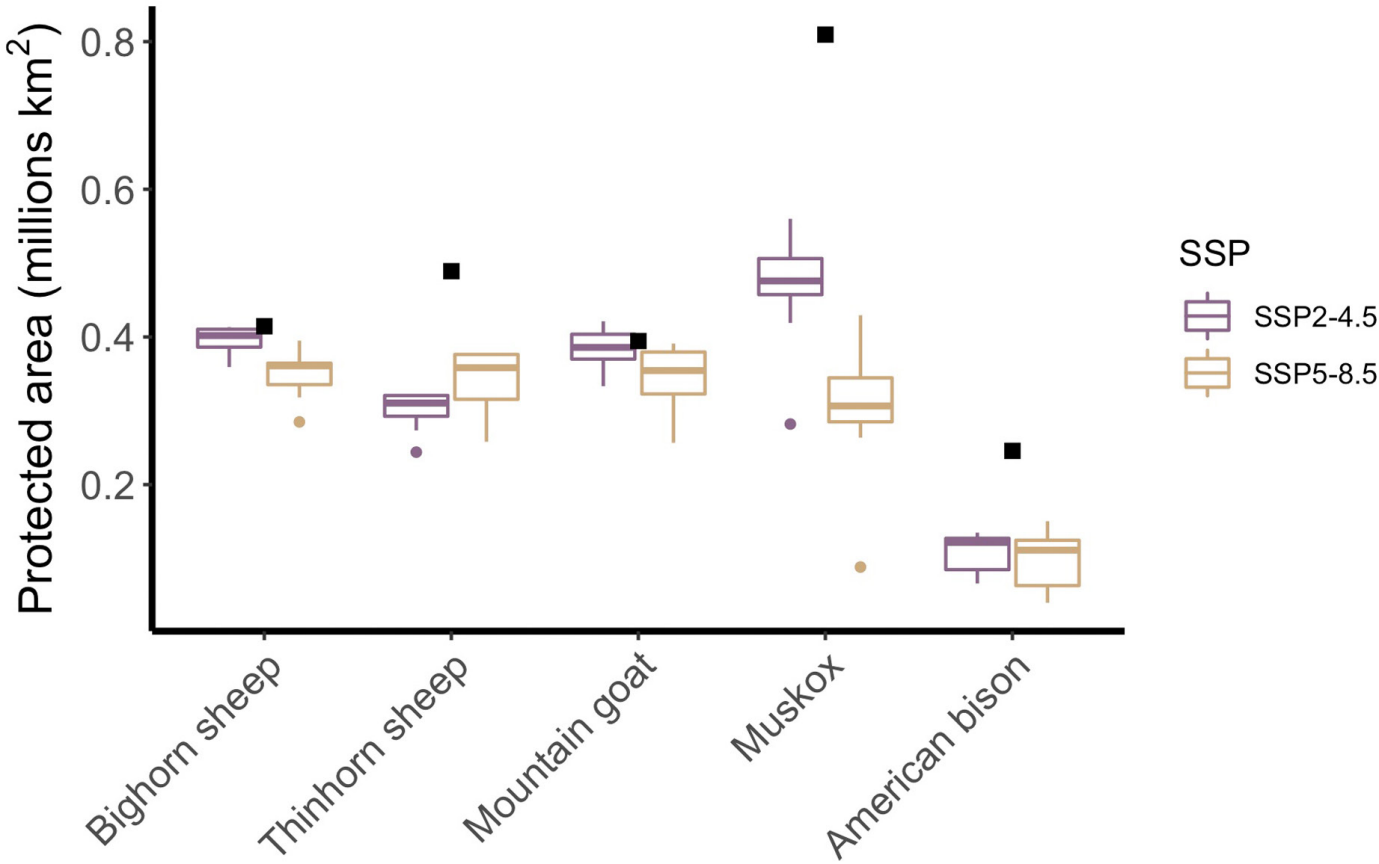
Protected area of modeled species distributions in millions of km^2^. Squares indicate the land area of modeled current distributions that fall within protected areas, and boxes illustrate land area for modeled future distributions within protected areas under projected conditions for 2081–2100 under SSP2–4.5 (purple) and SSP5–8.5 (tan).

Overlap between ENM projections and current protected areas varied among species, and future overlap is expected to vary by species as well (Figure 2). Whereas habitat of southerly montane species with minimal projected range shifts (bighorn sheep and mountain goats) is not projected to face a significant change in potential protected area, habitat of northerly species such as thinhorn sheep and muskoxen is projected to face a considerable reduction in potential protected area (38.6% and 43.1% of protected area for thinhorn sheep and muskoxen, respectively, under SSP2–4.5, and 30.5% and 62.9% under SSP5–8.5). Projected loss of potential protected area for bison followed a similar pattern (55.3% and 59.3% for SSP2–4.5 and SSP5–8.5, respectively). For the only obligate Arctic species, muskoxen, the projected reduction in potential protected area is considerably greater under the SSP5–8.5 scenario than under SSP2–4.5 (nearly 20% greater reduction in potential protected area under SSP5–8.5).

**FIGURE 3:**
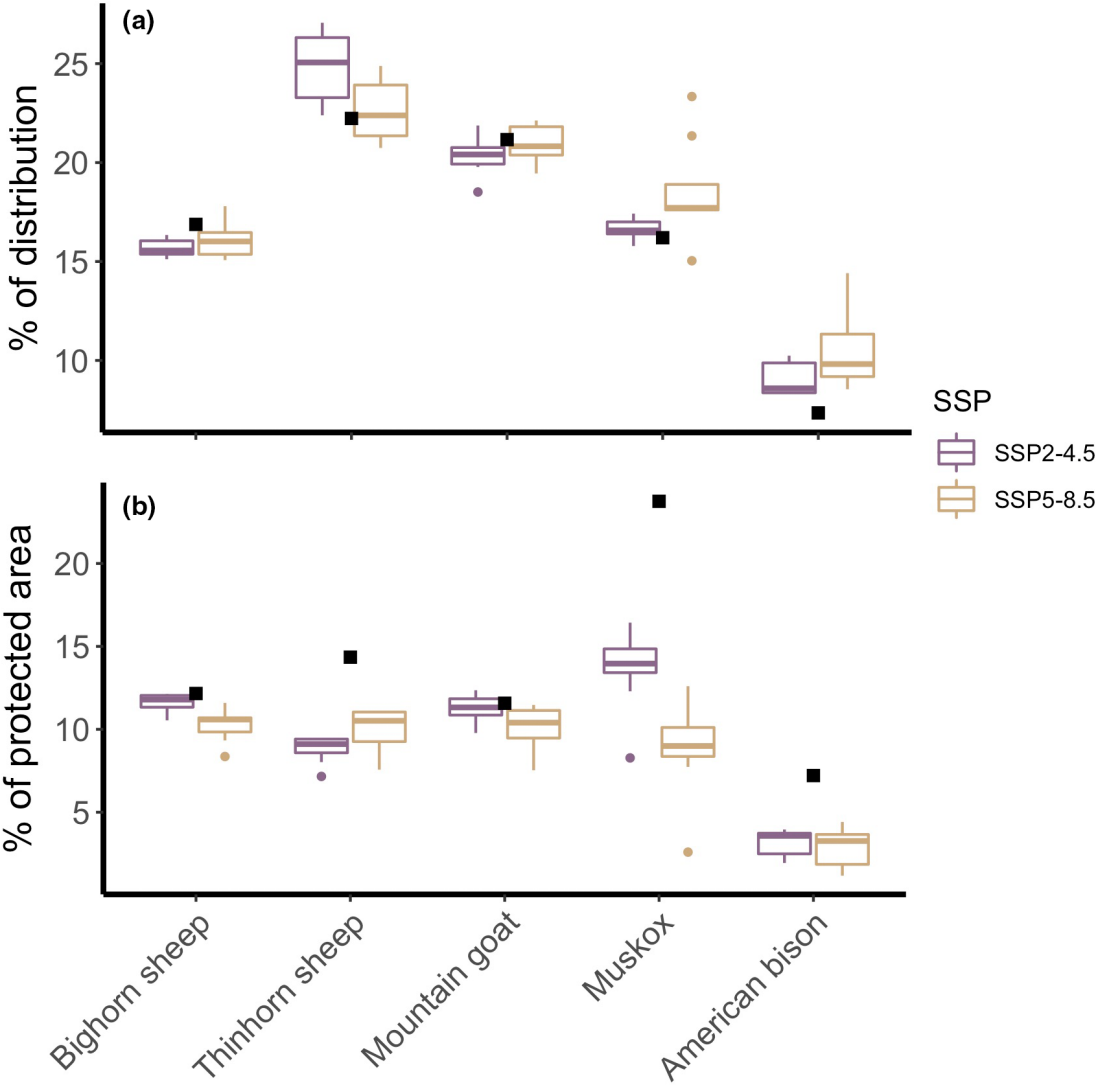
Potential protected area expressed as a percentage of potential species distributions (a) and as a percentage of currently protected area (b). In a, the proportion is calculated based on the percentage of each species, GCM, and SSP‐specific potential distribution that overlaps with protected areas. In b, the proportion is calculated based on the percentage of currently protected areas that overlap with each species, GCM, and SSP‐specific potential distribution. Black dots indicate current potential distribution estimates, purple boxes show variation in SSP2–4.5 scenarios across GCMs, and tan boxes show variation in SSP5–8.5 scenarios across GCMs.

The proportion of potential species distributions that overlaps with protected area and the proportion of protected area that overlaps with potential distributions reveal different patterns in potential protected area among the bovid species (Figure 3). Although approximately proportional loss of protected area relative to potential species distributions is projected across the five North American bovid species (indicated by overlapping current and projected estimates in Figure 3a), the percentage of currently protected area that is projected to feature environments characterized by bovid presence is projected to drop across SSPs for thinhorn sheep, muskox, and American bison (indicated by the marked reduction in fraction of protected area estimated for these species in Figure 3b).

## 4 DISCUSSION

We identified discordant projections by MaxEnt distribution models across Nearctic bovids. Inconsistent projections among species arose through two processes: unequal response by species to different topographic, land cover, and bioclimatic variables, and uneven projected environmental change across space. Projected potential habitat shifts in response to anticipated climate change are greatest for species at high latitudes, where observed warming outpaced change at lower latitudes, and is expected to continue to do so (Post, Steinman, & Mann, 2018; Post et al., 2019). Furthermore, for some species, the total area of potential protected space is projected to decrease more dramatically under the higher emissions scenario, SSP5–8.5. Shifts in climatically suitable habitat seem likely for other high latitude species, where effects of climate change are amplified.

Other species distribution modeling efforts corroborate the importance of human impacts, terrain, and land cover characteristics for ungulate distributions (Herrera‐Sánchez et al., 2020; Jenkins et al., 2020; Kuemmerle et al., 2012). To our knowledge, this is the first study to simultaneously explore future distributions of multiple North American bovids in the context of protected areas. However, modeling studies that employ different data sources and different scales of analysis have uncovered important relationships between bovid species and their environment that help contextualize our findings.

In a recent study, a MaxEnt model for desert bighorn sheep (*Ovis canadensis nelsoni*) was hindcast to investigate range dynamics during the mid‐Holocene (Gámez‐Brunswick & Rojas‐Soto, 2020). Although this subspecies occupies only a portion of the total range of bighorn sheep, the modeled current potential distribution of desert bighorn in that study largely mirrors the current potential distribution of bighorn sheep across the southwest United States predicted by our models. Importantly, our results suggest that potential habitat extends further northward along the American cordillera than either the hindcast model of desert bighorn or the actual current distribution of bighorn sheep (Brewer et al., 2014). The predicted presence of bighorn sheep through Yukon and Alaska likely relates to the similar life history requirements of the closely related thinhorn sheep (*Ovis dalli*), which inhabits these higher latitude regions of the cordillera. Indeed, among the selected predictor variables that were shared among bighorn and thinhorn sheep MaxEnt models, most univariate response curves were approximately comparable with shifted centers. Furthermore, a comparison of the two species' modeled distributions reveals considerable overlap north of British Colombia. It is possible that the lack of bighorn sheep at high latitudes stems from competitive exclusion by thinhorn sheep, or through fine‐scale environmental variation that was not evident at the scale of our study.

MaxEnt distribution models have also been used to examine spatial dynamics of muskoxen at local to regional, but not continental scales (Beumer et al., 2019; Jenkins et al., 2020; van Beest et al., 2021). In those applications, GPS collars and systematic human observations were used to identify environmental covariates underlying muskox distribution in Northeast Greenland and the Canadian Arctic. Across levels of analysis, elevation emerged as an important covariate of muskox distribution, following the same tendency of selection toward low elevations we found here (Beumer et al., 2019; Jenkins et al., 2020). Notably, our variable selection and model optimization process did not retain the same bioclimatic variables that were selected in one study using Worldclim2 data (van Beest et al., 2021), but that work included a subset of candidate predictor variables, used a coarser covariate resolution (20 km), and the study extent was limited to northeast Greenland, as opposed to our 6 km analysis of North America.

The results of this modeling study suggest a broader spatial range of present potential habitat than is realized for any of these five bovid species (Brewer et al., 2014; Côté & Festa‐Bianchet, 2003; Cuyler et al., 2020; Demarchi & Hartwig, 2004; Meagher, 1986). For example, predictions from the muskox model indicate that Southampton and Baffin Islands are within the potential distribution of muskox, yet that species is not known to live there. Overprediction of actual distributions may have resulted from the coarse nature of our predictor data (6 × 6 km pixels), limiting factors that we were not able to account for (e.g., predation, important forage species, or habitat fragmentation by non‐permeable barriers), or more complex responses to environmental variables than we allowed in our modeling design (such as absolute thermal tolerance thresholds or interactions among variables). Thus, the modeling results should be interpreted in the context of predicted change in environmental factors associated with bovid presence, rather than spatial redistribution of bovid species themselves.

The predictive ability of species distribution models is limited by the extent to which current predictor variables relate to the environment at the time of occurrence data collection, and the degree to which covariate forecasts represent future conditions. Worldclim data are least reliable in mountainous terrain, where fine‐scale complexity overwhelms broad geographic variation and in remote areas where only sparse meteorological records were available for model training (Fick & Hijmans, 2017; Hijmans et al., 2005). Furthermore, species distribution forecasts may be sensitive to inconsistent variation among modeled bioclimatological futures (Cerasoli et al., 2022) and uncertainty related to the underlying GCMs (Bedia et al., 2013; Foley, 2010). Finally, predicted future distributions rest upon assumptions about future change; for example, GCAM land use data incorporates no developments in urbanization through the end of the century, and modeled vegetation change stems only from land use impacts, as opposed to vegetation response to warming (Chen et al., 2020), which is complex (Myers‐Smith et al., 2020) and important for spatial dynamics of large herbivores (Tape et al., 2016).

Our model projections are based on relationships between observations of bovids and environmental factors where they were observed. In reality, drivers of range dynamics in large herbivores are complex and unlikely to relate directly to climatological variability. Instead, indirect effects of climate such as forage distribution and phenology, distribution of competitors and natural enemies, and frequency and severity of extreme weather events are likely to play important roles in changes in species distributions related to climate change (Creel et al., 2005; Parmesan et al., 2000; Ponti & Sannolo, 2022; Winnie et al., 2008). Historical relationships among humans and megafauna may drive patterns in species distribution, particularly if species are refugees from human exploitation (Cromsigt et al., 2012). The importance of human impacts was evident for several of the species we investigated; for example, fractional agriculture and urban cover were the second‐ and third‐most important variables in the thinhorn sheep model, which revealed strong patterns of selection against both cover types. Agriculture was the fifth‐most important variable in the bison model, which showed a weaker pattern of selection against urban cover. While agriculture and urban land cover did not emerge as important factors in other models, it is likely that a more precise land cover data product (in terms of both spatial resolution and cover type) would reveal significant human effects. For example, the Human Footprint Index (1 km resolution) may uncover fine‐scale impacts of light and infrastructure on current bovid distributions that we could not explore here (Venter et al., 2016), but a comparable forecast product is not currently available. Further, the ability of bovid populations to redistribute in future will be limited by not only available destination space, but also by barriers to movement (McInturff et al., 2020; Sawyer et al., 2013).

Conservation planning is sensitive to biases in species distribution models (Wilson et al., 2005), and we emphasize the need to incorporate multiple approaches and lines of evidence in planning future protected areas. Furthermore, although spatial priorities for protected areas increasingly rely on species distribution projections under climate change, they often ignore human response to climate change (Jones et al., 2016; Post & Brodie, 2015). Human influence on the landscape limits movements by animals, which may ultimately lead to the local exclusion of broad‐ranging migrants (Tucker et al., 2018). Other work on large bovids has emphasized the importance of anthropogenic influence on habitat suitability (Epps et al., 2005; Kuemmerle et al., 2010). We were unable to include movement barriers and some human impacts on species ranges, such as roads and fencing, tourism, and recreation. More precise estimates of future suitable habitat for large herbivores will become possible as forecasts of anthropogenic change across the landscape become clearer.

Most immediately, North American bovids contend with alteration of existing suitable habitat (Krausman & Bleich, 2013), limitations on movement between seasonal ranges (Courtemanch et al., 2017; Stoellinger et al., 2020), and introduction of zoonotic disease (Clifford et al., 2009). These threats are difficult to predict, and changes in their distribution and magnitude should be considered while crafting management and conservation plans. Of the protected areas that are already home to wild bovids, those which are expected to retain ecological and climatic characteristics that are associated with bovid presence may become especially important in the coming decades. As conservation planners make decisions about designation of new protected areas, it will be imperative to consider not just the future distribution of Nearctic bovids, but also future conditions for ecosystem services and human response to change (IPBES, 2019). Protected areas conserve ecosystem function, culturally important settings, recreational hotspots, and natural resources. However, if biodiversity, or the longevity of a particular species is the goal, future climatological conditions and their implication for the focal species and increased human access to remote regions should be a top consideration in the prioritization of protected lands.

## AUTHOR CONTRIBUTIONS

**Christian John:** Conceptualization (lead); formal analysis (lead); methodology (lead); software (lead); visualization (lead); writing – original draft (lead); writing – review and editing (equal). **Eric Post:** Conceptualization (supporting); writing – original draft (supporting); writing – review and editing (equal).

## CONFLICT OF INTEREST

None.

## Supporting information


Table S1

Table S2‐S3

Figure S1

## Data Availability

All data used in this work are publicly available, and code to reproduce findings is available at https://www.github.com/JepsonNomad/NearcticBovidae.
