# Supplementary material for: Projected bioclimatic distributions in Nearctic Bovidae signal the potential for reduced overlap with protected areas
Source: Ecol Evol. 2022 Aug 11;12(8):e9189. doi: 10.1002/ece3.9189 (PMC9366586; doi:10.1002/ece3.9189)
Supplement: Supplementary file 1 — Table S1 [file ECE3-12-e9189-s003.docx]

**Supplementary materials S1**

ODMAP protocol for ENM reporting:

| **ODMAP section** | **ODMAP subsection** | **ODMAP elements** |
| --- | --- | --- |
| **Overview** | **Authorship** | Christian John and Eric Post. Department of Wildlife, Fish, and Conservation Biology at the University of California, Davis. Contact: cjohn@ucdavis.edu. Projected bioclimatic distributions in Nearctic Bovidae signal the potential for reduced overlap with protected areas. doi available upon acceptance for publication. |
|  | **Model objective** | Predict changes in overlap between North American bovid species and currently protected areas. The target output is forecasted species distributions for 5 North American bovid species |
|  | **Taxon** | Bighorn sheep (*Ovis canadensis*), Thinhorn sheep (*Ovis dalli*), Mountain goat (*Oreamnos americanus*), Muskox (*Ovibos moschatus*), and American bison (*Bison bison*) |
|  | **Location** | Terrestrial North America and its islands |
|  | **Scale of analysis** | Spatial extent designed using political boundaries and includes Central and North America (xmin = 171.79°W; xmax = 12.20°W; ymin = 7.22°N; ymax = 83.65°N). Raster data were resampled to 6kmx6km pixels in Albers projection (+proj=aea +lat_0=40 +lon_0=-96 +lat_1=20 +lat_2=60 +x_0=0 +y_0=0 +datum=NAD83 +units=m +no_defs). "Present" climate data were from 1970-2000; Future climate data for 2081-2100. |
|  | **Biodiversity data overview** | Observation data (presence-only) collected from GBIF records; predictor variables generated from WorldClim v2.1 (<https://www.worldclim.org/>), GCAM Demeter land use (https://data.pnnl.gov/group/nodes/dataset/13192), and North America Elevation 1-kilometer resolution GRID (https://www.sciencebase.gov/catalog/item/4fb5495ee4b04cb937751d6d). Topography data were treated as fixed, not varying between model fitting and prediction. |
|  | **Type of predictors** | WorldClim bioclimatic variables, Demeter land use/land cover variables, and elevation/topography data. |
|  | **Hypotheses** | We expected northward shifts and elevational contractions between historical and future modeled distributions, adjusting the representation of bioclimatic ranges in currently protected areas. |
|  | **Assumptions** | We assume niche conservatism, error-free predictors, full knowledge of important predictors, observation independence, spp.-environment equilibrium, unbiased observations, and stationarity in model predictive ability. |
|  | **SDM algorithms** | MaxEnt v3.4.3 was used with presence and 10000 background observations. Model results were compared with an identical procedure with 5000 background points and found to be in general agreement (r > 0.7 for all species). |
|  | **Model workflow** | For each species, MaxEnt models were fit to a 60% training, 20% validation, and 20% testing subset of occurrence data using checkerboard cross-validation. Variable importance was assessed with permutation and jackknife tests. After cross-validation, full models were fit for each species. Full models were then used to project 2081-2100 suitable habitat using CMIP6 climate projections. |
|  | **Software, codes, and data** | R version v3.6.1 "Action of the Toes", dismo v1.3.3, and Maxent v3.4.1 were used. Code for data access, cleaning, and model fit and predictions available at <https://github.com/JepsonNomad/NA_Bovidae_SDM>. Data available for bioclimatic data at <https://www.worldclim.org/>; for landuse data <https://data.pnnl.gov/group/nodes/dataset/13192>; for Elevation data <https://www.sciencebase.gov/catalog/item/4fb5495ee4b04cb937751d6d>; and for GBIF occurrences <https://doi.org/10.15468/dl.burd8t > |
| **Data** | **Biodiversity data** | Taxon names described above. Data were analyzed at the species level, using the GBIF system with species ID's 2441119, 2441118, 2441151, 2441108, 2441176 (respectively). GBIF occurrence data (<https://www.gbif.org/>) were accessed 5 May 2022 by way of the R package `rgbif` and the GBIF API. Data were filtered to retain only points on North America and Greenland, and to remove occurrences where latitude and longitude were not available, or where the listed observation coordinates were in a different country from the listed observation country. Points with observation dates prior to 1980 were also removed. Preserved specimens and fossil records were removed. Finally, several additional GBIF records were manually removed for other idiosyncratic reasons (Table 1). Occurrence records were thinned to a 6km radius, following the resolution of predictor data, to avoid duplicate sampling.   \| Table 1. GBIF records that were manually removed from the SDM analyses, and their reasons for removal. \| \| \| \| --- \| --- \| --- \| \| **Species** \| **GBIF ID** \| **Reason for removal** \| \| Bighorn sheep \| 922490545 \| Denali (probably thinhorn) \| \| Bighorn sheep \| 1019041688 \| Recorded at zoo in Lansing Michigan \| \| American bison \| 2596125567 \| Camp Pendleton, introduced \| \| American bison \| 1850921137 \| Camp Pendleton, introduced \| \| American bison \| 2381410738 \| Camp Pendleton, introduced \| \| American bison \| 2269206648 \| Camp Pendleton, introduced \| \| American bison \| 3079682696 \| Camp Pendleton, introduced \| \| American bison \| 3415452210 \| Camp Pendleton, introduced \| \| American bison \| 3457071522 \| Camp Pendleton, introduced \| \| American bison \| 2631191303 \| San Fransisco Zoo \| \| American bison \| 2631191308 \| San Fransisco Zoo \| \| American bison \| 2631191306 \| Golden Gate Park \| \| American bison \| 1893583451 \| Weird Mexico loc’s \| \| American bison \| 1893583408 \| Weird Mexico loc’s \| \| American bison \| 3456432067 \| Ranch near Santa Ysabel \|   Occurrence map for the 5 North American bovid species: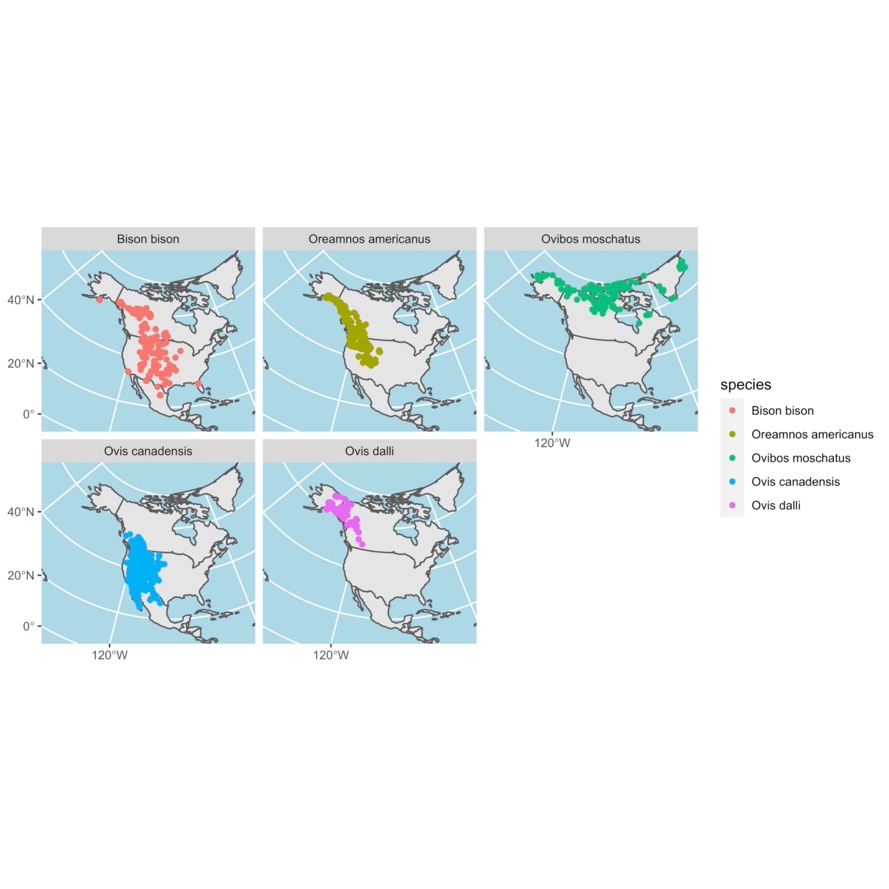 |
|  | **Data partitioning** | We used a 60% subset of the original data to train the model for each species, and a 20% subset for validation. The remaining 20% were used for model testing. GBIF occurrence records, thinning, training, validation, and testing data partitioning can be recreated using the code on our GitHub repository <https://github.com/JepsonNomad/NA_Bovidae_SDM>. |
|  | **Predictor variables** | Historic and future (CMIP6) bioclimatic variables were accessed at the WorldClim (v2.1) website (<https://worldclim.org/>) on 2 March 2021. We accessed 2.5m global data but cropped the dataset to North America and resampled to 6km pixels in an Albers Equal Area projection (see above) using bilinear interpolation with GDAL. GCAM Demeter data were accessed from the PNNL website (<https://data.pnnl.gov/group/nodes/dataset/13192>) on 3 June 2021 and resampled as above. Terrain data were accessed from <https://www.sciencebase.gov/catalog/item/4fb5495ee4b04cb937751d6d> on 26 July 2018 and resampled as above. Terrain ruggedness index (TRI) were also calculated using default GDAL settings. |
|  | **Transfer data for projection** | All available CMIP6 model forecasts under SSP2-4.5 and SSP5-8.5 in the WorldClim v2 dataset were used for the period 2081-2100. Data were accessed at the WorldClim (v2.1) website <https://worldclim.org/>, and resampled following the same protocol as predictor variables used for model training. Differences between historical data and forecasts were visualized by converting temperature data to °C (original data in °C*10), centering based on historical data, and averaging across CMIP6 model projections. |
| **Model** | **Variable pre-selection** | All predictor variables were centered using the mean value of the resampled historic data. CMIP6 forecast data were centered according to the historic center values. GCAM Demeter data were condensed into single scenarios from CMIP6 models because the individual models available did not correspond with those from Worldclim. Elevation and terrain ruggedness index were also included. These variables remained unchanged between fitting the model on historic data and predicting with CMIP6 forecasts. |
|  | **Multicollinearity** | Variables were removed through an iterative data-driven modeling process (Vignali et al 2020). For each species, Maxent models were fit with checkerboard1 cross-validation and allowing only linear and quadratic feature classes. When variables were highly correlated (r > 0.7), the variable with the most explanatory power (identified using a leave-one-out jackknife test) was retained. This process was repeated for the resulting model until correlations among predictor variables fell below 0.7. Model transfers were fit using CMIP6 forecasts for the bioclimatic layers, GCAM Demeter forecasts for the landcover data, and unchanged terrain data. |
|  | **Model settings** | We used clamping and limited predictions to areas with positive MESS values (Elith et al 2010) to avoid extrapolation of conditions outside the range of the training data. 20% of input data were held for model testing. We allowed only linear and quadratic feature types to be used in model fitting, and then used a genetic algorithm to identify the best set of model hyperparameters (Vignali et al 2020). |
|  | **Model estimates** | Variable importance was determined through data permutation and jackknife tests (Vignali et al 2020). |
|  | **Model selection** | We did not use ensemble modeling to generate estimates, and instead relied on the final model generated after selection of model hyperparameters and variable reduction. |
|  | **Non-independence correction** | Non-independence is an inherent feature of many citizen science datasets, and we acknowledge that points used to train the models described here likely feature both spatial and temporal autocorrelation. We used spatial thinning of occurrence to account for spatial autocorrelation and bias grids to generate background data. |
|  | **Threshold selection** | Thresholding was used in order to translate the 8 CMIP model-based ENM projections into consensus plots, so that each model was equally weighted as 0=absent, 1=present. Sensitivity and specificity were maximized to determine the species-specific presence/absence threshold, although we compared this metric to a threshold based on equal sensitivity and specificity in Figure 1 (finding similar results). In preliminary modeling steps, we found this threshold selection technique related closely to the maximized true skill statistic. |
| **Assessment** | **Performance statistics** | Training and testing AUC were compared to assess model training. Models were evaluated using a 20% partition of fully-withheld testing data. |
|  | **Plausibility check** | We performed plausibility checks for each species-specific model by plotting predicted presence for the baseline (1970-2000) predictor conditions, and found that models were generally predictive of known current species distributions (but see discussion on pattern of overprediction). Plausibility was also ascertained using marginal and sole-predictor response curves of important variables. |
| **Prediction** | **Prediction output** | Prediction outputs were defined using thresholding to differentiate potential presence (1) and absence (0), which we interpreted as "suitable" and "unsuitable" habitat, respectively. Across CMIP6 models, prediction outputs for future climate scenarios resulted in a discrete model consensus surface with the minimum possible value being 0 (no models predict presence) and maximum possible value being 8 (all models agree on presence). |
|  | **Uncertainty quantification** | Uncertainty in future conditions was assessed using multi-model consensus, outlined above. Boundary conditions – such as non-analogue climates – were accounted for using clamping and MESS grid during predictions, described above.  Future climate MESS grids (positive values only shown):   \|  \| SSP 2-4.5 \| SSP 5-8.5 \| \| --- \| --- \| --- \| \| BCC-CSM2-MR \| 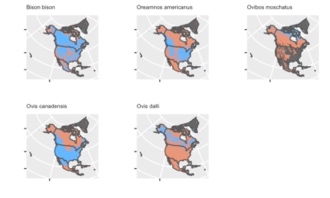 \| 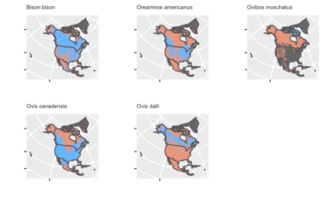 \| \| CanESM5 \| 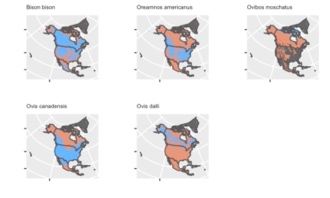 \| 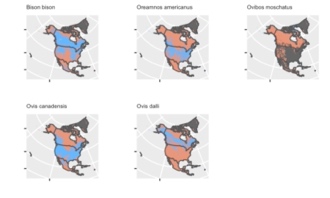 \| \| CNRM-CM6-1 \| 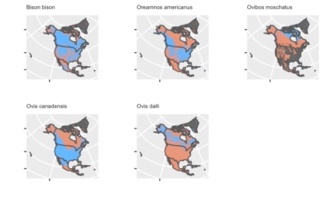 \| 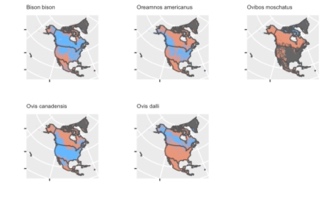 \| \| CNRM-ESM2-1 \| 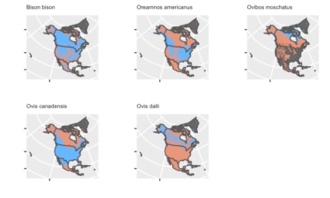 \| 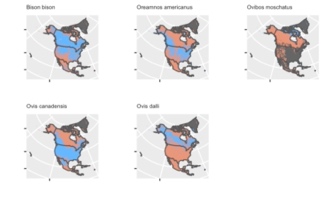 \| \| IPSL-CM6A-LR \| 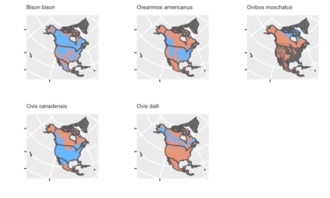 \| 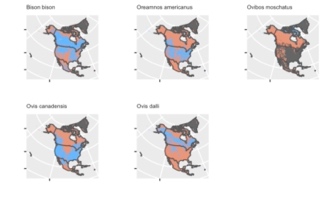 \| \| MIROC-ES2L \| 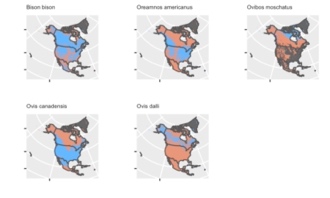 \| 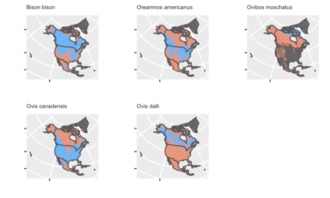 \| \| MIROC6 \| 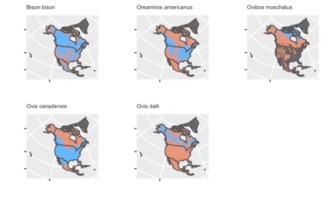 \| 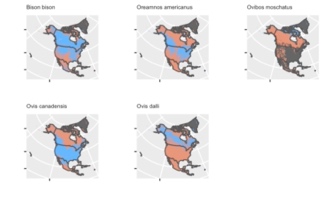 \| \| MRI-ESM2-0 \| 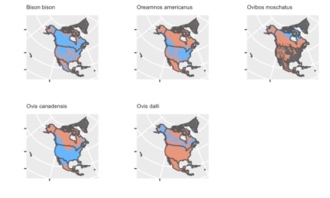 \| 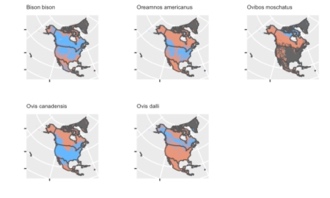 \| |
