# Supplementary material for: Projected bioclimatic distributions in Nearctic Bovidae signal the potential for reduced overlap with protected areas
Source: Ecol Evol. 2022 Aug 11;12(8):e9189. doi: 10.1002/ece3.9189 (PMC9366586; doi:10.1002/ece3.9189)

**Supplementary Materials S2**

Table 1. Modeled current and projected mean elevation of each Nearctic bovid species, in meters above sea level.


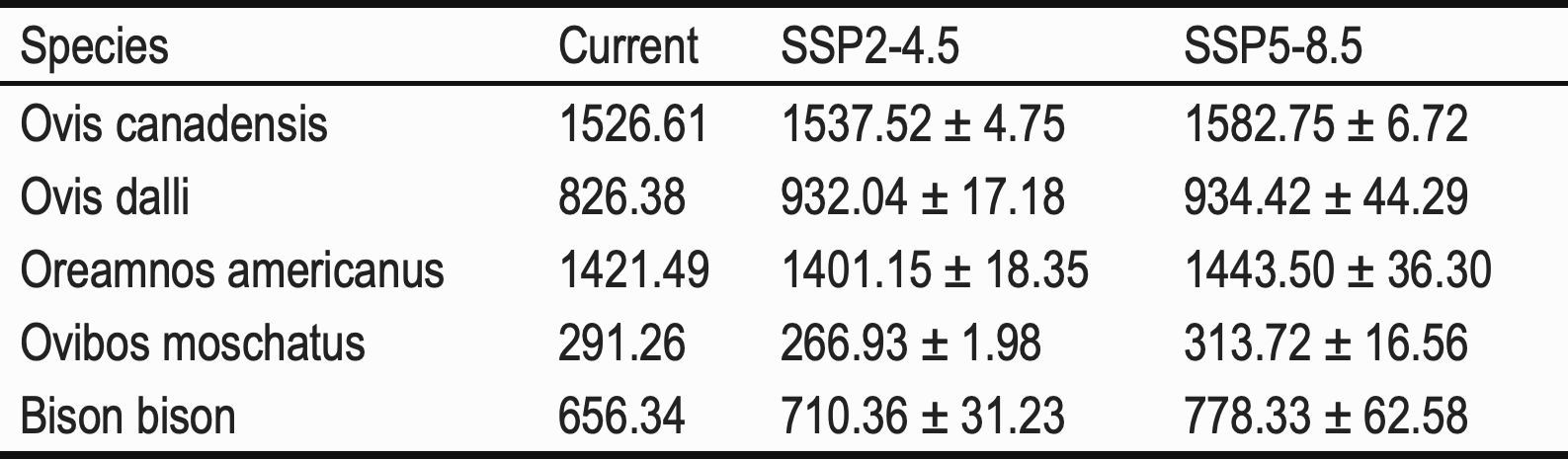


Table 2. Modeled current and projected mean latitude of each Nearctic bovid species, shown in degrees N.


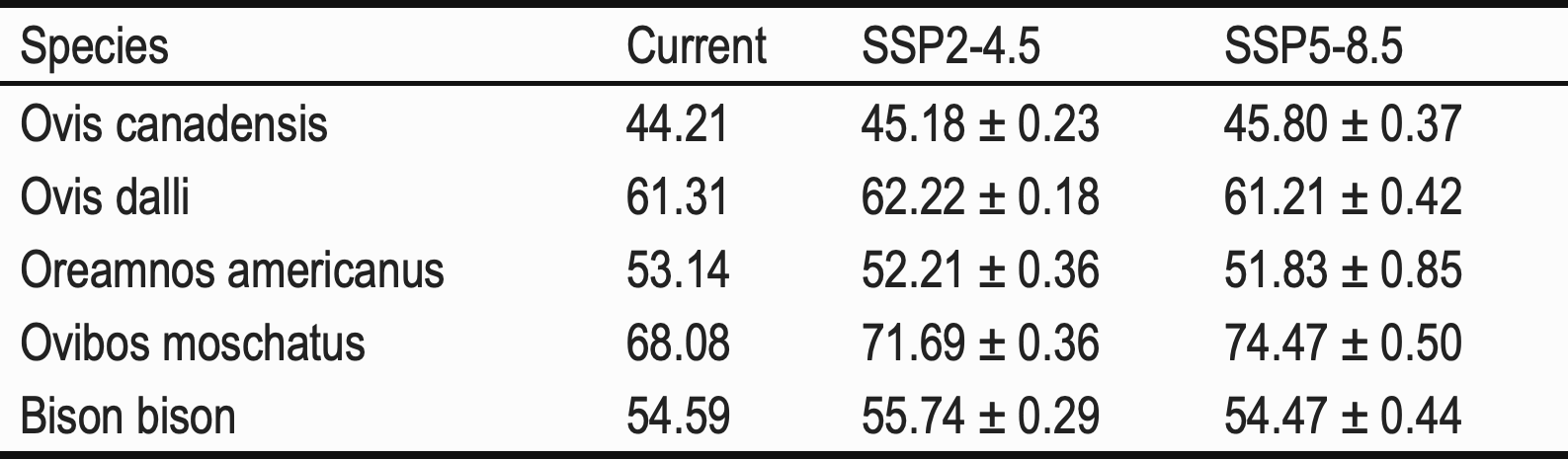

Supplement: Supplementary file 2 — Table S2‐S3 [file ECE3-12-e9189-s002.docx]
