# Supplementary material for: Projected bioclimatic distributions in Nearctic Bovidae signal the potential for reduced overlap with protected areas
Source: Ecol Evol. 2022 Aug 11;12(8):e9189. doi: 10.1002/ece3.9189 (PMC9366586; doi:10.1002/ece3.9189)
Supplement: Supplementary file 3 — Figure S1 [file ECE3-12-e9189-s001.docx]

Supplementary materials S3. The following are Receiver-Operator Characteristic curves, variable importance tables, and univariate response curves for the 5 most important variables for each species. Univariate response curves can be interpreted as the response of habitat suitability to that variable on its own, without considering other variables.

**Bighorn sheep**

| 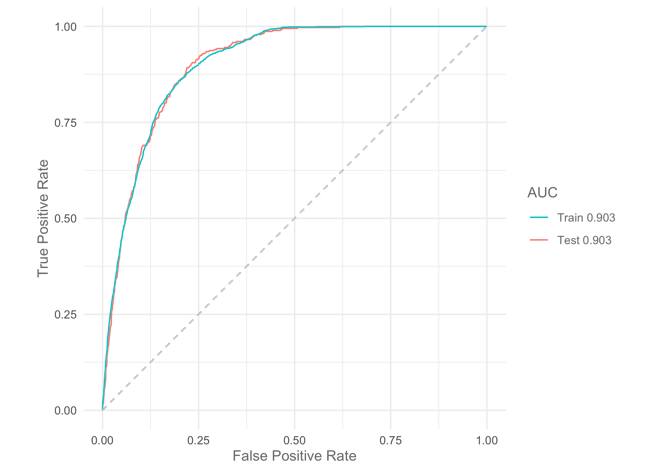 | \| **Variable** \| **Permutation_importance** \| **sd** \| \| --- \| --- \| --- \| \| TRI \| 41.0 \| 0.003 \| \| Biocl_12 \| 36.5 \| 0.004 \| \| Biocl_04 \| 11.6 \| 0.004 \| \| C3_gra_arc \| 3.4 \| 0.002 \| \| Biocl_08 \| 2.1 \| 0.001 \| \| treeTemperate \| 1.4 \| 0.001 \| \| Biocl_15 \| 1.1 \| 0.001 \| \| treeBoreal \| 1.0 \| 0.000 \| \| C4_gra \| 0.9 \| 0.001 \| \| Biocl_02 \| 0.7 \| 0.001 \| \| C3_gra \| 0.2 \| 0.000 \| |
| --- | --- | --- | --- | --- | --- | --- | --- | --- | --- | --- | --- | --- | --- | --- | --- | --- | --- | --- | --- | --- | --- | --- | --- | --- | --- | --- | --- | --- | --- | --- | --- | --- | --- | --- | --- | --- | --- |
| 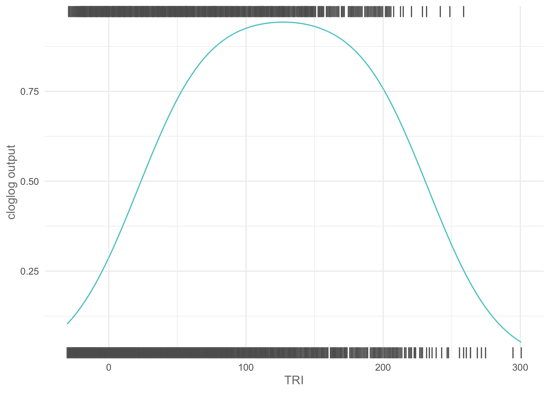 | 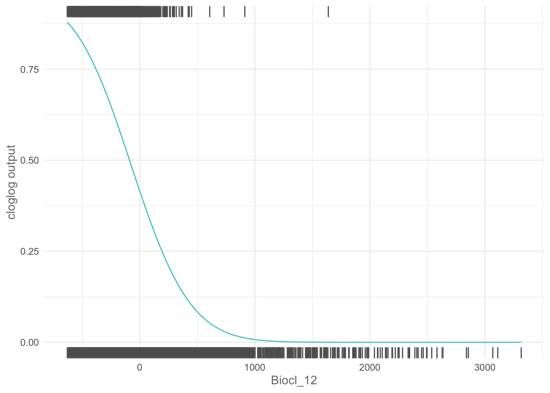 |
| 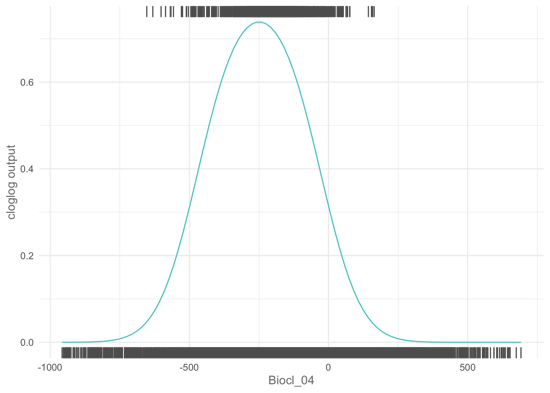 | 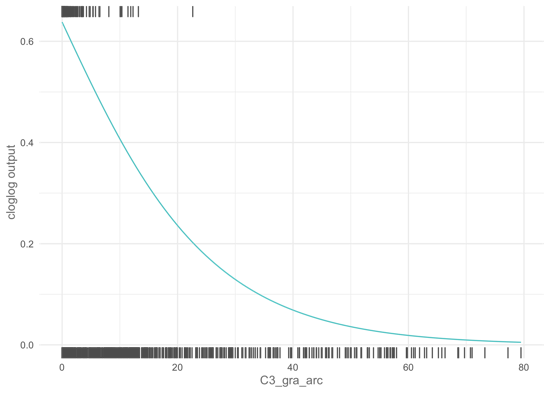 |
| 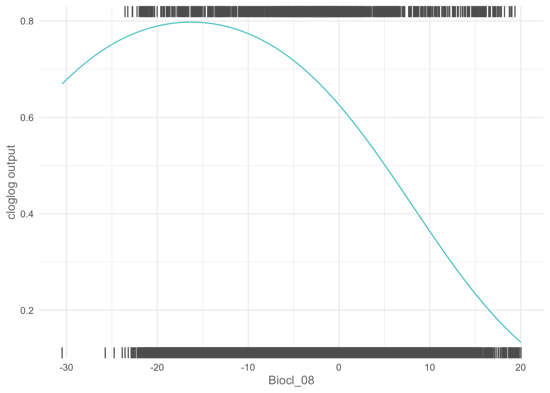 | TRI: Terrain ruggedness index  Biocl_12: Annual precipitation  Biocl_04: Temperature seasonality  C3_gra_arc: C3 Arctic grasses fractional cover  Biocl_08: Mean temperature of wettest quarter |

**Thinhorn sheep**

| 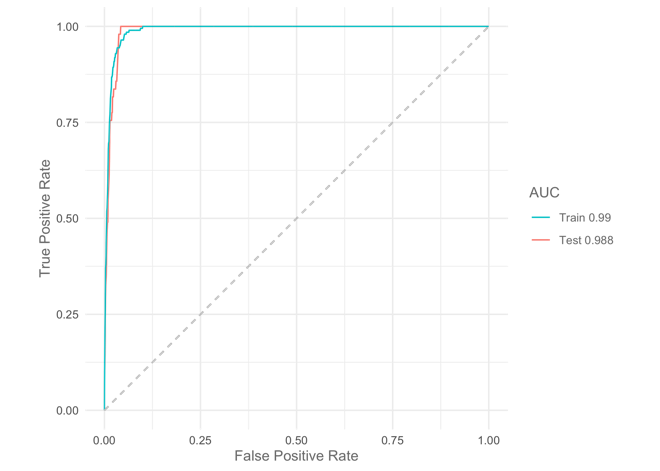 | \| **Variable** \| **Permutation_importance** \| **sd** \| \| --- \| --- \| --- \| \| Biocl_03 \| 61.1 \| 0.014 \| \| AGR \| 13.0 \| 0.008 \| \| Urban \| 8.8 \| 0.007 \| \| Biocl_19 \| 6.8 \| 0.010 \| \| treeTemperate \| 6.7 \| 0.003 \| \| TRI \| 2.7 \| 0.001 \| \| treeBoreal \| 0.5 \| 0.000 \| \| C3_gra_arc \| 0.2 \| 0.000 \| \| C3_gra \| 0.1 \| 0.000 \| |
| --- | --- | --- | --- | --- | --- | --- | --- | --- | --- | --- | --- | --- | --- | --- | --- | --- | --- | --- | --- | --- | --- | --- | --- | --- | --- | --- | --- | --- | --- | --- | --- |
| 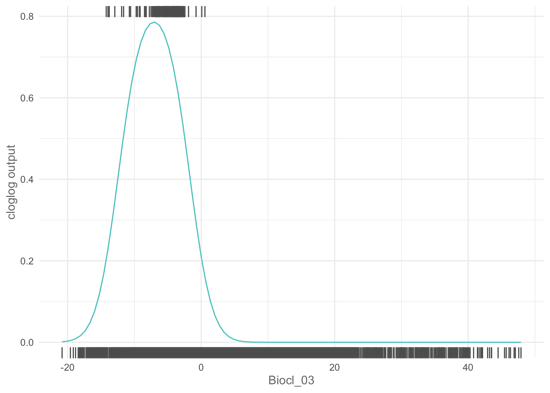 | 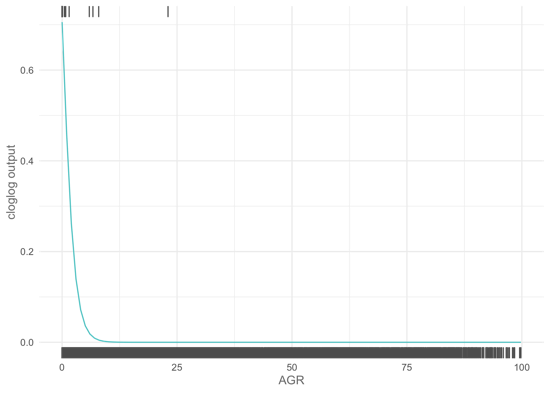 |
| 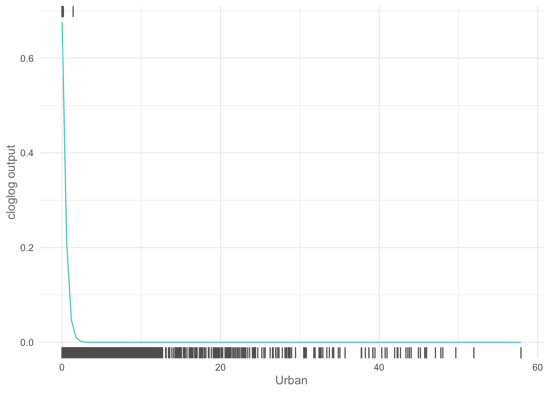 | 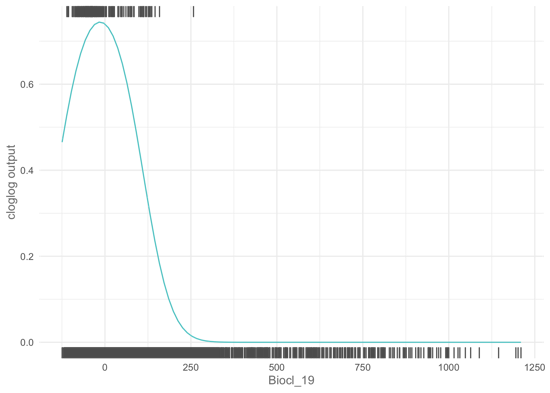 |
| 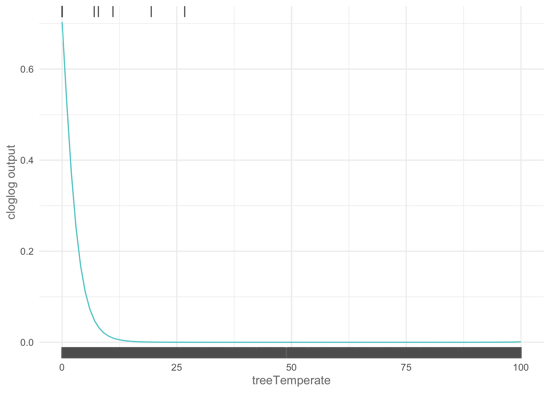 | Biocl_03: Isothermality  AGR: Agriculture fractional cover  Urban: Urban fractional cover  Biocl_19: Precipitation of coldest quarter  treeTemperate: Temperate tree fractional cover |

**Mountain goat**

| 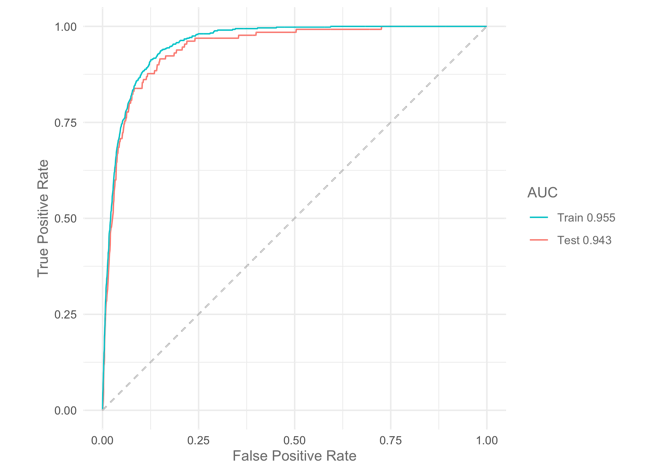 | \| **Variable** \| **Permutation_importance** \| **sd** \| \| --- \| --- \| --- \| \| TRI \| 63.6 \| 0.005 \| \| Biocl_03 \| 24.2 \| 0.007 \| \| treeTemperate \| 3.8 \| 0.001 \| \| BDS_tem \| 2.6 \| 0.002 \| \| Biocl_18 \| 2.0 \| 0.001 \| \| C4_gra \| 1.3 \| 0.001 \| \| DEM \| 0.9 \| 0.000 \| \| treeBoreal \| 0.6 \| 0.000 \| \| Biocl_15 \| 0.4 \| 0.000 \| \| C3_gra \| 0.4 \| 0.000 \| \| Biocl_19 \| 0.2 \| 0.000 \| \| BDS_bor \| 0.1 \| 0.000 \| |
| --- | --- | --- | --- | --- | --- | --- | --- | --- | --- | --- | --- | --- | --- | --- | --- | --- | --- | --- | --- | --- | --- | --- | --- | --- | --- | --- | --- | --- | --- | --- | --- | --- | --- | --- | --- | --- | --- | --- | --- | --- |
| 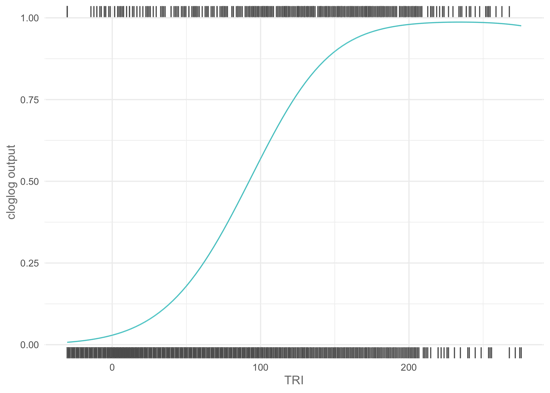 | 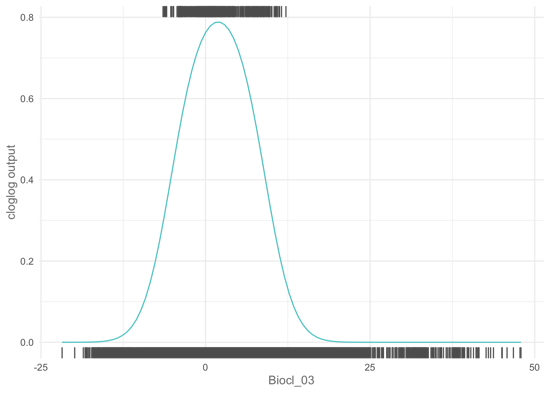 |
| 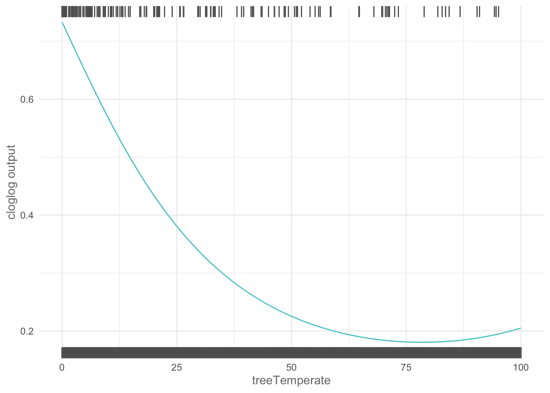 | 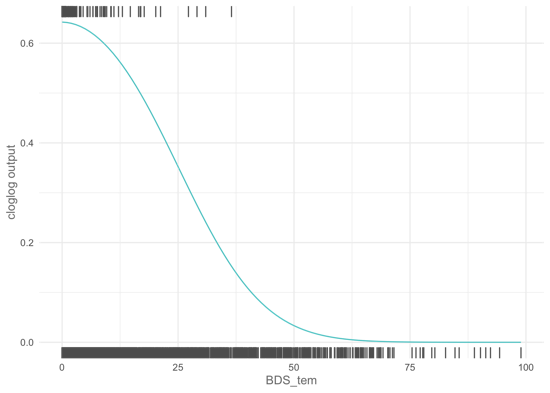 |
| 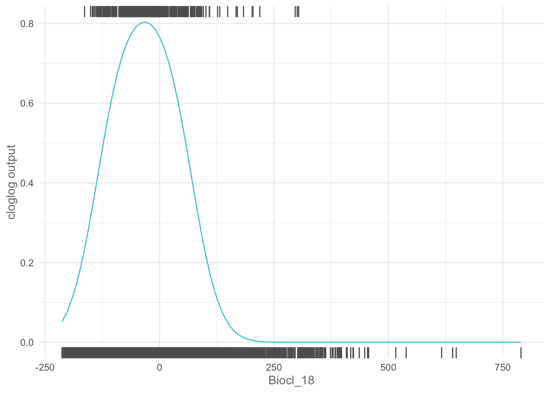 | TRI: Terrain ruggedness index  Biocl_03: Isothermality  treeTemperate: Temperate tree fractional cover  BDS_tem: Broadleaf deciduous shrub fractional cover  Biocl_18: Precipitation of warmest quarter |

**Muskox**

| 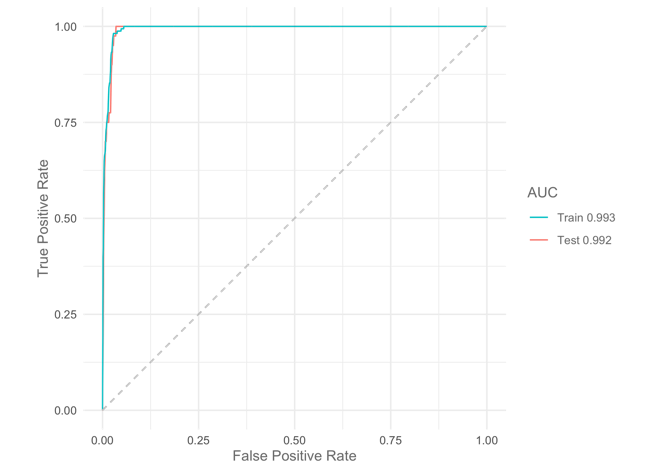 | \| **Variable** \| **Permutation_importance** \| **sd** \| \| --- \| --- \| --- \| \| Biocl_10 \| 70.6 \| 0.022 \| \| DEM \| 17.1 \| 0.006 \| \| treeTemperate \| 7.4 \| 0.005 \| \| Biocl_14 \| 1.3 \| 0.002 \| \| Biocl_18 \| 0.9 \| 0.000 \| \| treeBoreal \| 0.9 \| 0.001 \| \| Biocl_04 \| 0.8 \| 0.001 \| \| TRI \| 0.7 \| 0.001 \| \| C3_gra \| 0.2 \| 0.000 \| |
| --- | --- | --- | --- | --- | --- | --- | --- | --- | --- | --- | --- | --- | --- | --- | --- | --- | --- | --- | --- | --- | --- | --- | --- | --- | --- | --- | --- | --- | --- | --- | --- |
| 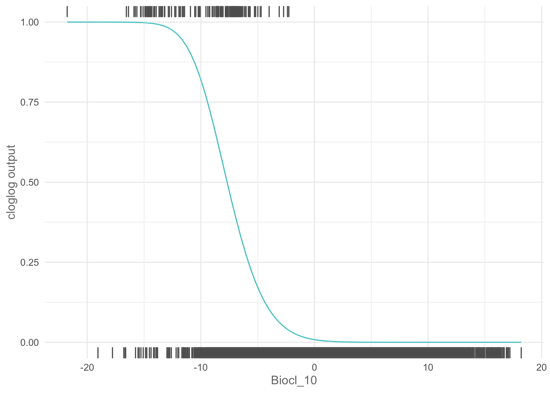 | 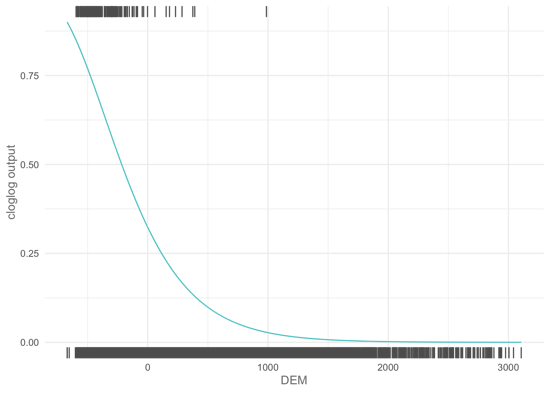 |
| 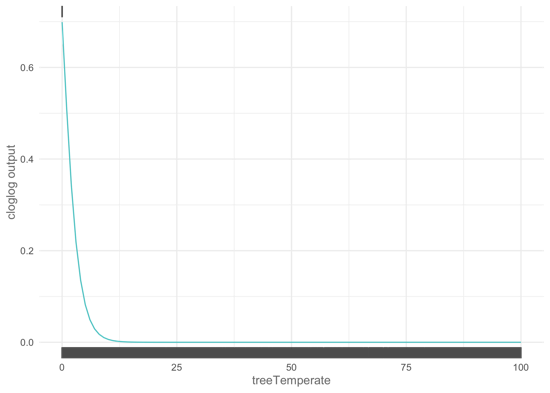 | 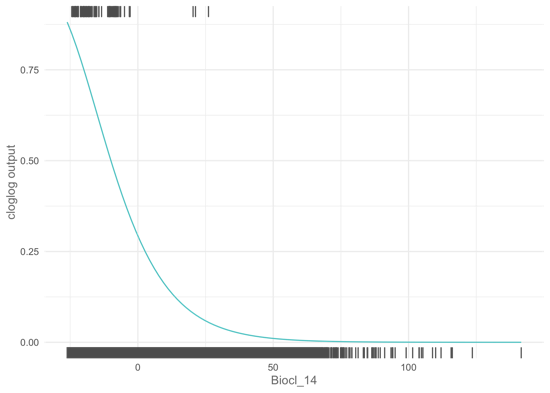 |
| 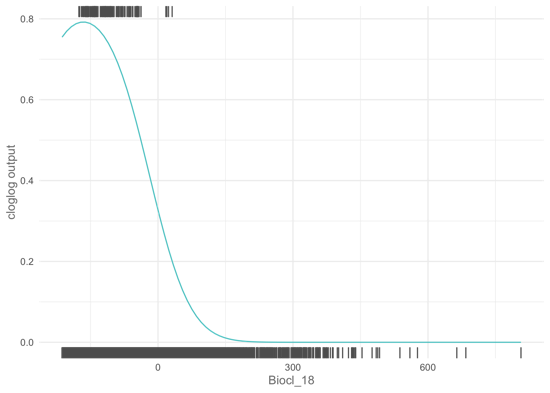 | Biocl_10: Mean temperature of warmest quarter  DEM: Elevation  treeTemperate: Temperate tree fractional cover  Biocl_14: Precipitation of driest month  Biocl_18: Precipitation of warmest quarter |

**American bison**

| 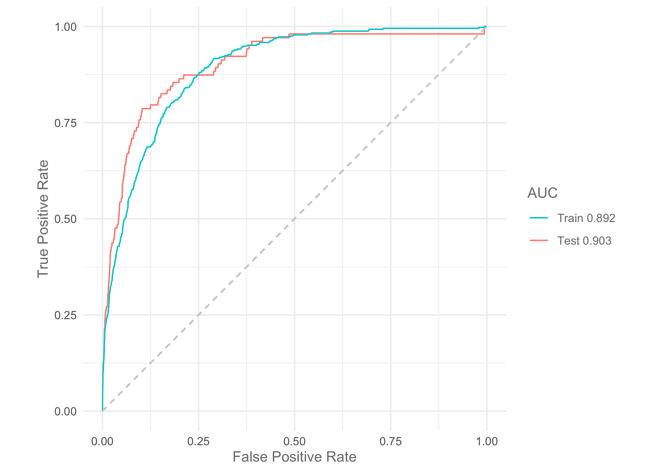 | \| **Variable** \| **Permutation_importance** \| **sd** \| \| --- \| --- \| --- \| \| Biocl_19 \| 26.3 \| 0.009 \| \| Biocl_18 \| 23.3 \| 0.006 \| \| Biocl_08 \| 13.1 \| 0.003 \| \| Biocl_02 \| 10.9 \| 0.005 \| \| AGR \| 9.6 \| 0.004 \| \| treeBoreal \| 6.0 \| 0.002 \| \| treeTemperate \| 3.0 \| 0.003 \| \| TRI \| 2.7 \| 0.002 \| \| BDS_tem \| 2.1 \| 0.002 \| \| C3_gra_arc \| 1.5 \| 0.002 \| \| C4_gra \| 1.0 \| 0.001 \| \| DEM \| 0.5 \| 0.001 \| \| Urban \| 0.0 \| 0.001 \| |
| --- | --- | --- | --- | --- | --- | --- | --- | --- | --- | --- | --- | --- | --- | --- | --- | --- | --- | --- | --- | --- | --- | --- | --- | --- | --- | --- | --- | --- | --- | --- | --- | --- | --- | --- | --- | --- | --- | --- | --- | --- | --- | --- | --- |
| 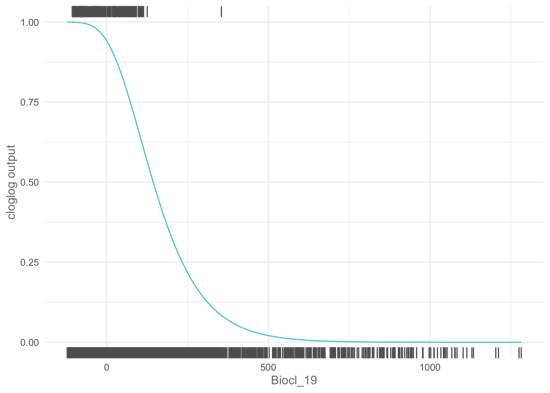 | 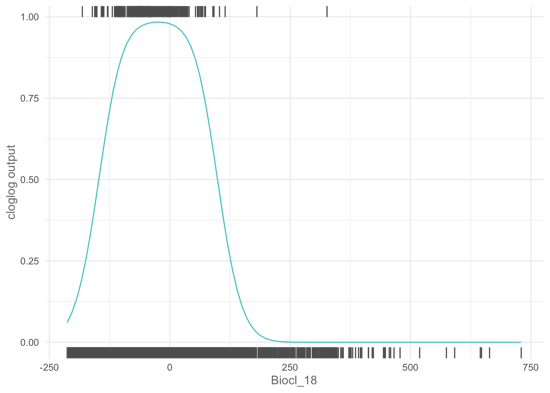 |
| 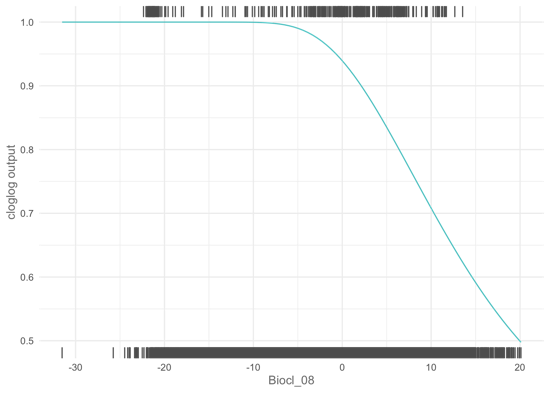 | 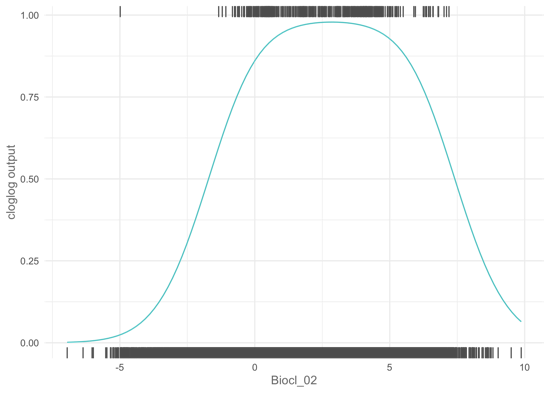 |
| 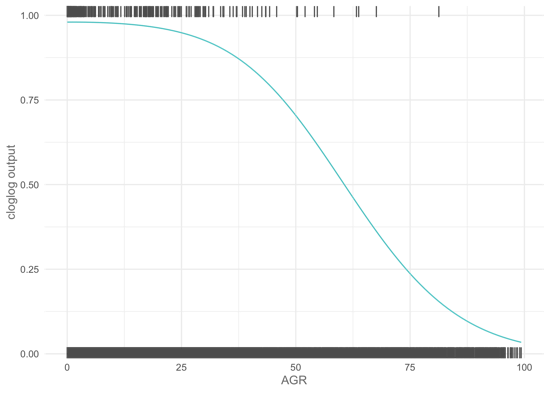 | Biocl_19: Precipitation of coldest quarter  Biocl_18: Precipitation of warmest quarter  Biocl_08: Mean temperature of wettest quarter  Biocl_02: Mean diurnal range  AGR: Agriculture fractional cover |
